# ALCOHOL CONSUMPTION AND ABUSIVE/NEGLECTFUL BEHAVIORS AMONG FAMILY CAREGIVERS OF PEOPLE LIVING WITH DEMENTIA

**DOI:** 10.1093/geroni/igad104.2018

**Published:** 2023-12-21

**Authors:** Jessica Hernandez Chilatra, Wesley Browning, Mustafa Yildiz, Vicki Winstead, Carolyn Pickering

**Affiliations:** The University of Alabama at Birmingham, Birmingham, Alabama, United States; The University of Texas Health Science Center - Houston, Houston, Texas, United States; University of Alabama at Birmingham, Birmingham, Alabama, United States; University of Alabama at Birmingham, Birmingham, Alabama, United States; University of Alabama at Birmingham, Birmingham, Alabama, United States

## Abstract

Despite its suspected role in elder abuse and neglect (EAN), little is known about alcohol use disorders (AUD) among family caregivers of people with Alzheimer’s disease and related dementia (ADRD). As part of a micro-longitudinal study in which caregivers completed 21 daily surveys about their caregiving experiences, we examined the effects of daily drinking behaviors and screening positive for hazardous drinking (based on the Alcohol Use Disorder Identification Test (AUDIT-C)) on the daily risk of EAN. A generalized linear mixed model with days (n = 9513) nested within caregivers (N=453) was used to evaluate the relationships between caregivers’ alcohol consumption patterns and the odds of using an abusive and/or neglectful behavior. Caregivers’ alcohol consumption on a given day increases the odds of using physical abuse (OR=2.19; 95% CI=1.12–4.28; p=.022), psychological abuse (OR=1.40; 95% CI=1.01–1.94; p=.041) and neglect (OR=1.48; 95% CI=1.17–1.86; p=.001) that same day. Screening positive for AUD significantly increases the odds of a caregiver’s use of neglect on a given day (OR=2.43, 95% CI=1.52–3.88, p=<.001), but not physical or psychological abuse. There were no interaction effects between AUD and daily drinking, meaning AUD increases the risk of neglect independent of daily drinking behavior. Interventions to manage AUD may reduce the risk of neglect among ADRD caregivers experiencing hazardous drinking. Interventions focused on daily drinking behaviors are needed to reduce the general risk of EAN among ADRD caregivers. Findings show that subpopulations exist within ADRD caregivers who engage in EAN behaviors that would benefit from different targeted interventions.

